# Supplementary material for: Quantifying In-Host Quasispecies Evolution
Source: Int J Mol Sci. 2023 Jan 9;24(2):1301. doi: 10.3390/ijms24021301 (PMC9867078; doi:10.3390/ijms24021301)
Supplement: Supplementary file 1 [file ijms-24-01301-s001.zip › ijms-2094920-supplementary.pdf]

# Quantifying In-Host Quasispecies Evolution

Josep Gregori [1,*], Marta Ibañez-Lligoña [1,2,3] and Josep Quer [1,2,3]

[1] Liver Diseases-Viral Hepatitis, Liver Unit, Vall d'Hebron Institut de Recerca (VHIR), Vall d'Hebron Barcelona Hospital Campus, Passeig Vall d'Hebron 119-129, 08035 Barcelona, Spain
[2] Centro de Investigación Biomédica en Red de Enfermedades Hepáticas y Digestivas (CIBERehd), Instituto de Salud Carlos III, Av. Monforte de Lemos, 3-5, 28029 Madrid, Spain
[3] Biochemistry and Molecular Biology Department, Universitat Autònoma de Barcelona (UAB), Campus de la UAB, Plaça Cívica, 08193 Bellaterra, Spain
* Correspondence: josep.gregori@vhir.org or josep.gregori@gmail.com

**Abstract:** What takes decades, centuries or millennia to happen with a natural ecosys-tem, it takes only days, weeks or months with a replicating viral quasispecies in a host, especially when under treatment. Some methods to quantify the evolution of a quasispecies are introduced and discussed, along with simple simulated examples to help in the interpretation and understanding of the results. The proposed methods treat the molecules in a quasispecies as indi-viduals of competing species in an ecosystem, where the haplotypes are the competing species, and the evolution of the system is quantified by monitor-ing changes in haplotype frequencies. The correlation between the proposed indices is also discussed, and the R code used to generate the simulations, the data and the plots is provided. The virtues of the proposed indices are finally shown on a clinical case.

**Keywords:** quasispecies evolution; distributions similarity; quasispecies fitness partition; viral treatment; mutagenesis

# Supplementary materials

# A Supplementary figures

## A.1 Distribution overlap

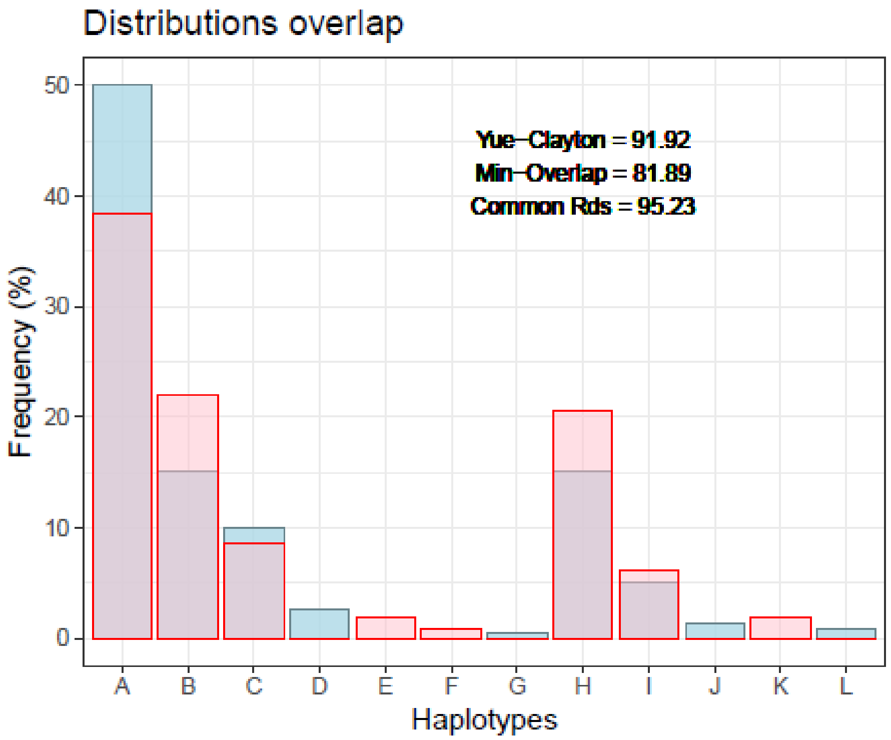


Figure S1: Montserrat plot illustrating distribution overlap.

### A.2 Histograms of simulated values

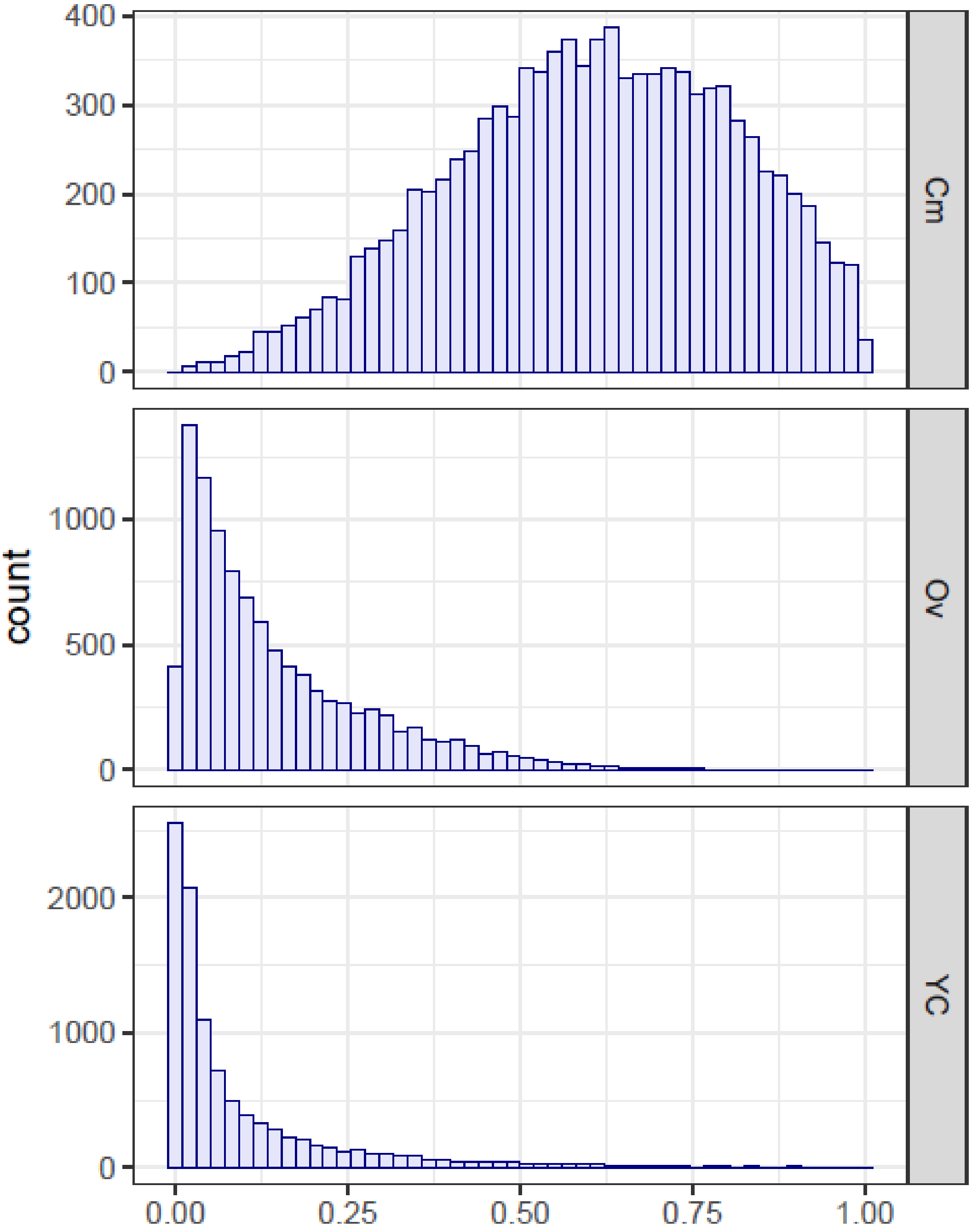


Figure S2: Histograms of values of similarity for the simulated pairs of quasispecies.

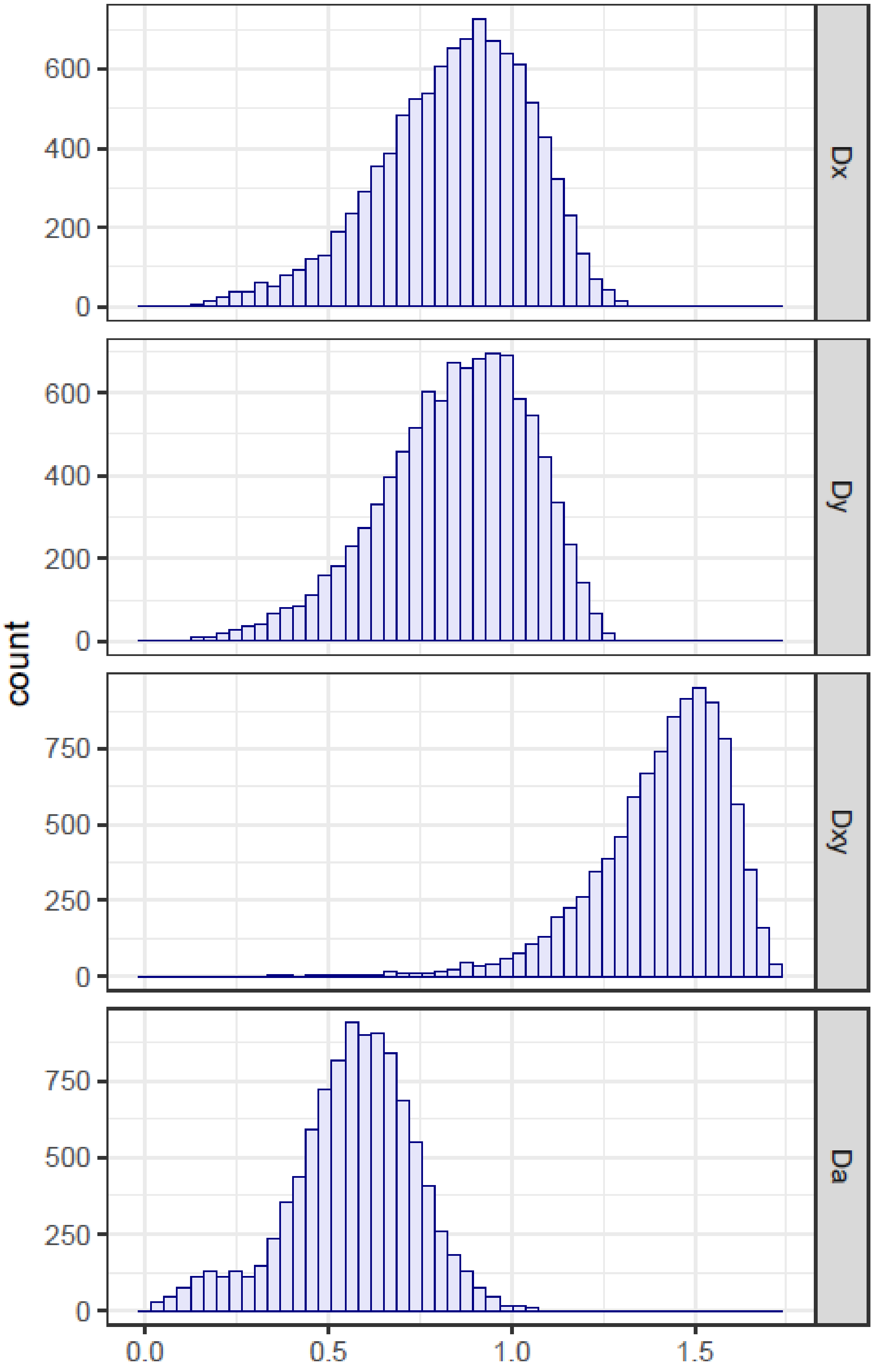


Figure S3: Histograms of values of nucleotide diversity and genetic distance for the simulated pairs of quasispecies.

### A.3 Tables and figures of selected quasispecies pairs

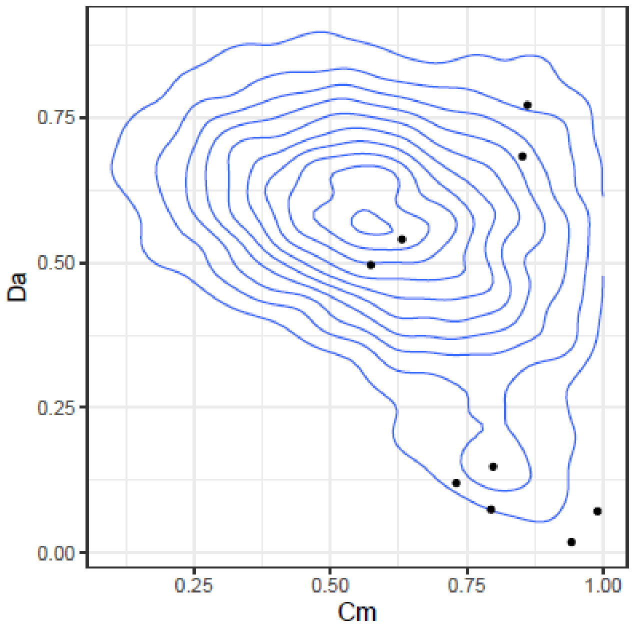


Figure S4: Selected pairs plotted on the $C_m$ and $D_A$ density plot.

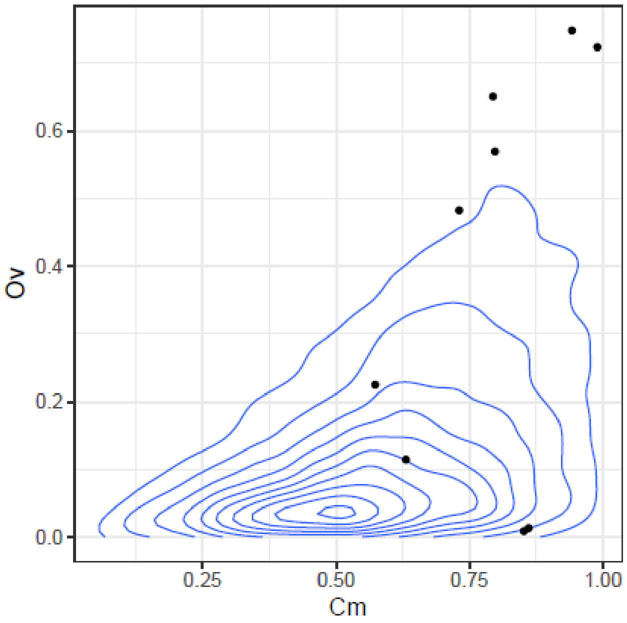


Figure S5: Selected pairs plotted on the $C_m$ and $O_v$ density plot.

| Hpl | nA | nB | pA | pB | Commons | Overlap |
|---|---|---|---|---|---|---|
| A | 5230 | 3808 | 52.30 | 38.08 | 45.19 | 38.08 |
| B | 3453 | 3059 | 34.53 | 30.59 | 32.56 | 30.59 |
| C | 528 | 981 | 5.28 | 9.81 | 7.55 | 5.28 |
| D | 355 | 15 | 3.55 | 0.15 | 1.85 | 0.15 |
| E | 233 | 0 | 2.33 | 0.00 | 0.00 | 0.00 |
| F | 85 | 0 | 0.85 | 0.00 | 0.00 | 0.00 |
| G | 56 | 90 | 0.56 | 0.90 | 0.73 | 0.56 |
| H | 39 | 0 | 0.39 | 0.00 | 0.00 | 0.00 |
| I | 8 | 4 | 0.08 | 0.04 | 0.06 | 0.04 |
| J | 7 | 402 | 0.07 | 4.02 | 2.05 | 0.07 |
| K | 4 | 0 | 0.04 | 0.00 | 0.00 | 0.00 |
| L | 1 | 842 | 0.01 | 8.42 | 4.22 | 0.01 |
| M | 0 | 614 | 0.00 | 6.14 | 0.00 | 0.00 |
| N | 0 | 21 | 0.00 | 0.21 | 0.00 | 0.00 |
| O | 0 | 163 | 0 | 1.63 | 0 | 0 |
| P | 0 | 2 | 0 | 0.02 | 0 | 0 |

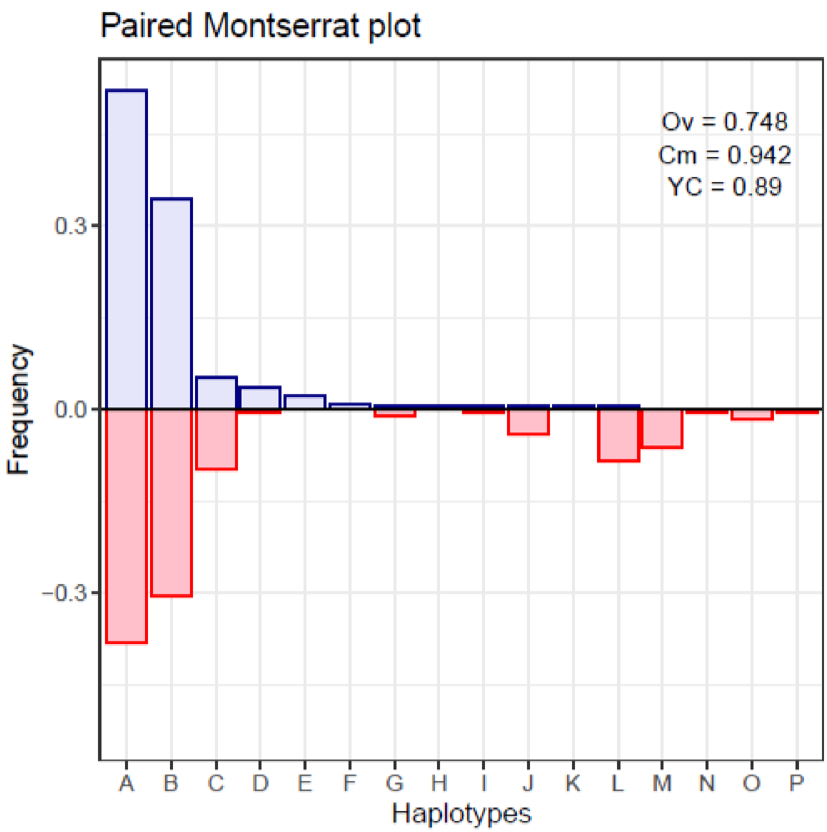


Figure S6: Simulated pair of index 4213.

| Hpl | nA | nB | pA | pB | Commons | Overlap |
|---|---|---|---|---|---|---|
| A | 3666 | 3142 | 36.66 | 31.42 | 34.04 | 31.42 |
| B | 2794 | 3899 | 27.94 | 38.99 | 33.47 | 27.94 |
| C | 1081 | 2541 | 10.81 | 25.41 | 18.11 | 10.81 |
| D | 909 | 2 | 9.09 | 0.02 | 4.56 | 0.02 |
| E | 766 | 79 | 7.66 | 0.79 | 4.23 | 0.79 |
| F | 474 | 115 | 4.74 | 1.15 | 2.95 | 1.15 |
| G | 265 | 2 | 2.65 | 0.02 | 1.34 | 0.02 |
| H | 22 | 5 | 0.22 | 0.05 | 0.14 | 0.05 |
| I | 13 | 25 | 0.13 | 0.25 | 0.19 | 0.13 |
| J | 6 | 0 | 0.06 | 0.00 | 0.00 | 0.00 |
| K | 3 | 0 | 0.03 | 0.00 | 0.00 | 0.00 |
| L | 1 | 0 | 0.01 | 0.00 | 0.00 | 0.00 |
| M | 0 | 145 | 0.00 | 1.45 | 0.00 | 0.00 |
| N | 0 | 14 | 0.00 | 0.14 | 0.00 | 0.00 |
| O | 0 | 33 | 0 | 0.33 | 0 | 0 |

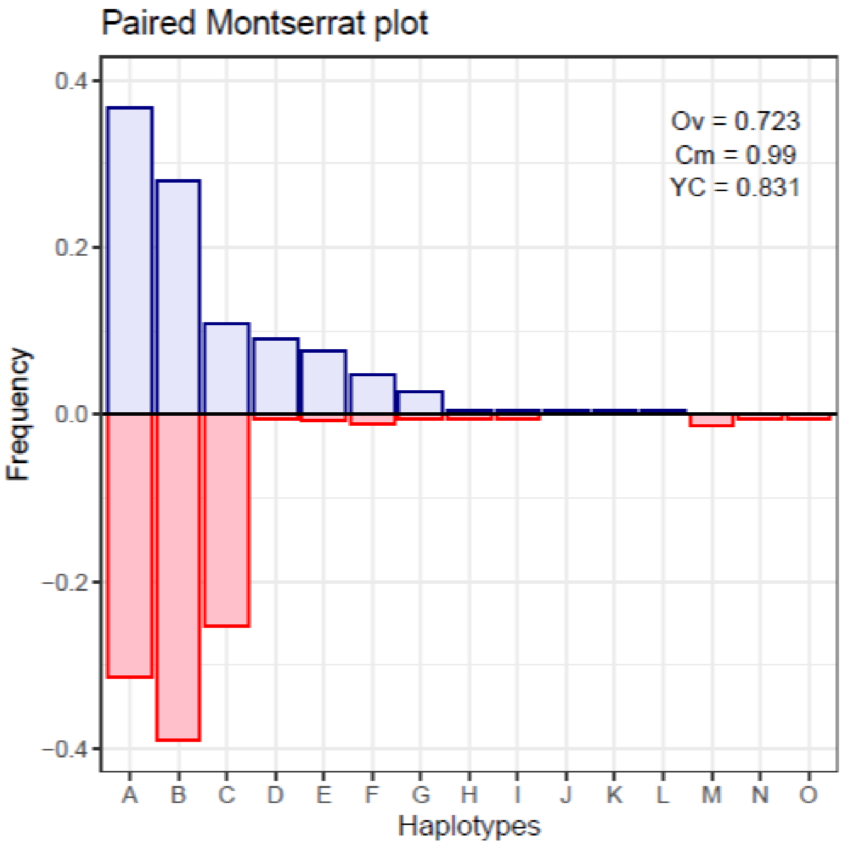


Figure S7: Simulated pair of index 7426.

| Hpl | nA | nB | pA | pB | Commons | Overlap |
|---|---|---|---|---|---|---|
| A | 4453 | 15 | 44.53 | 0.15 | 22.34 | 0.15 |
| B | 2834 | 4 | 28.34 | 0.04 | 14.19 | 0.04 |
| C | 1149 | 4 | 11.49 | 0.04 | 5.77 | 0.04 |
| D | 849 | 0 | 8.49 | 0.00 | 0.00 | 0.00 |
| E | 371 | 0 | 3.71 | 0.00 | 0.00 | 0.00 |
| F | 157 | 0 | 1.57 | 0.00 | 0.00 | 0.00 |
| G | 65 | 2377 | 0.65 | 23.77 | 12.21 | 0.65 |
| H | 47 | 6144 | 0.47 | 61.44 | 30.96 | 0.47 |
| I | 38 | 0 | 0.38 | 0.00 | 0.00 | 0.00 |
| J | 34 | 0 | 0.34 | 0.00 | 0.00 | 0.00 |
| K | 1 | 121 | 0.01 | 1.21 | 0.61 | 0.01 |
| L | 1 | 7 | 0.01 | 0.07 | 0.04 | 0.01 |
| M | 0 | 92 | 0.00 | 0.92 | 0.00 | 0.00 |
| N | 0 | 309 | 0.00 | 3.09 | 0.00 | 0.00 |
| O | 0 | 18 | 0 | 0.18 | 0 | 0 |
| P | 0 | 898 | 0 | 8.98 | 0 | 0 |
| Q | 0 | 11 | 0 | 0.11 | 0 | 0 |

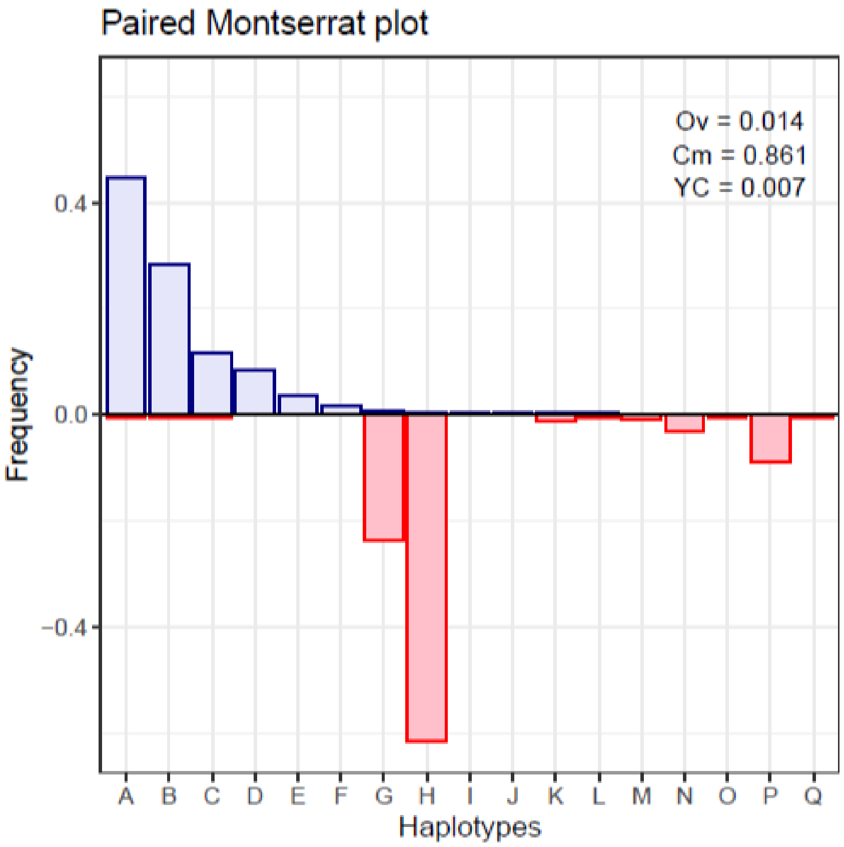


Figure S8: Simulated pair of index 774.

| Hpl | nA | nB | pA | pB | Commons | Overlap |
|---|---|---|---|---|---|---|
| A | 4360 | 1 | 43.60 | 0.01 | 21.81 | 0.01 |
| B | 3299 | 4 | 32.99 | 0.04 | 16.52 | 0.04 |
| C | 1473 | 0 | 14.73 | 0.00 | 0.00 | 0.00 |
| D | 604 | 0 | 6.04 | 0.00 | 0.00 | 0.00 |
| E | 89 | 0 | 0.89 | 0.00 | 0.00 | 0.00 |
| F | 72 | 0 | 0.72 | 0.00 | 0.00 | 0.00 |
| G | 48 | 2070 | 0.48 | 20.70 | 10.59 | 0.48 |
| H | 30 | 3076 | 0.30 | 30.76 | 15.53 | 0.30 |
| I | 13 | 0 | 0.13 | 0.00 | 0.00 | 0.00 |
| J | 9 | 4142 | 0.09 | 41.42 | 20.76 | 0.09 |
| K | 2 | 0 | 0.02 | 0.00 | 0.00 | 0.00 |
| L | 1 | 1 | 0.01 | 0.01 | 0.01 | 0.01 |
| M | 0 | 411 | 0.00 | 4.11 | 0.00 | 0.00 |
| N | 0 | 1 | 0.00 | 0.01 | 0.00 | 0.00 |
| O | 0 | 6 | 0 | 0.06 | 0 | 0 |
| P | 0 | 15 | 0 | 0.15 | 0 | 0 |
| Q | 0 | 264 | 0 | 2.64 | 0 | 0 |
| R | 0 | 9 | 0 | 0.09 | 0 | 0 |

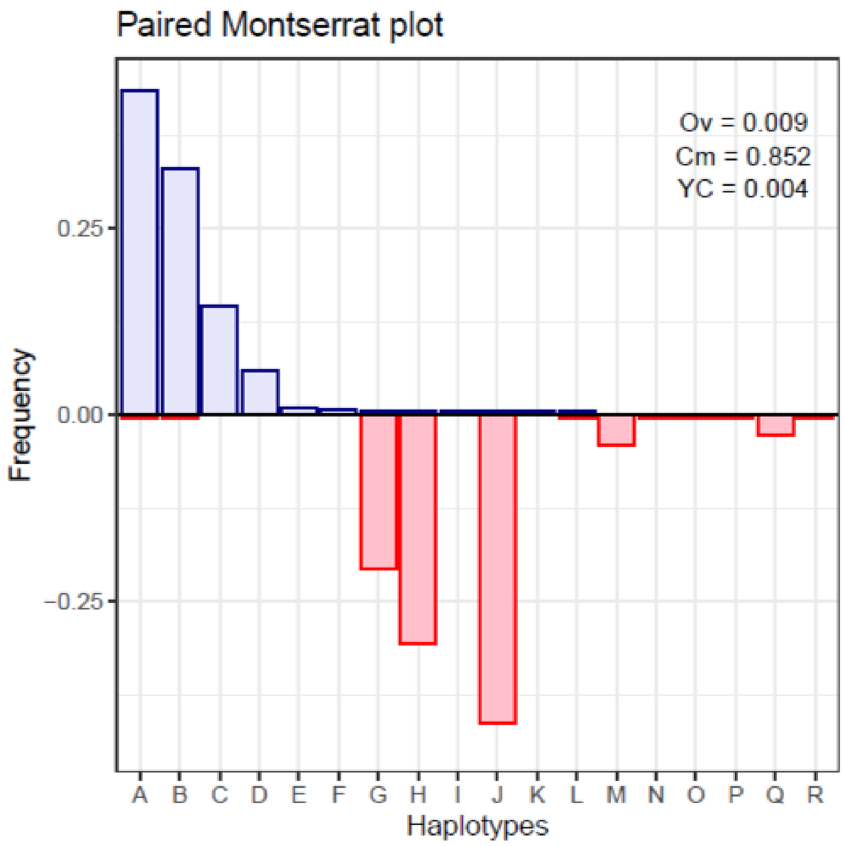


Figure S9: Simulated pair of index 5463.

| Hpl | nA | nB | pA | pB | Commons | Overlap |
|---|---|---|---|---|---|---|
| A | 1140 | 0 | 34.19 | 0.00 | 0.00 | 0.00 |
| B | 701 | 132 | 21.02 | 3.95 | 12.49 | 3.95 |
| C | 548 | 1424 | 16.43 | 42.73 | 29.58 | 16.43 |
| D | 361 | 50 | 10.83 | 1.51 | 6.17 | 1.51 |
| E | 291 | 0 | 8.73 | 0.00 | 0.00 | 0.00 |
| F | 145 | 0 | 4.36 | 0.00 | 0.00 | 0.00 |
| G | 58 | 2 | 1.75 | 0.05 | 0.90 | 0.05 |
| H | 30 | 12 | 0.90 | 0.35 | 0.63 | 0.35 |
| I | 29 | 6 | 0.87 | 0.18 | 0.53 | 0.18 |
| J | 22 | 0 | 0.67 | 0.00 | 0.00 | 0.00 |
| K | 8 | 0 | 0.23 | 0.00 | 0.00 | 0.00 |
| L | 1 | 474 | 0.03 | 14.21 | 7.12 | 0.03 |
| M | 0 | 77 | 0.00 | 2.31 | 0.00 | 0.00 |
| N | 0 | 1124 | 0.00 | 33.72 | 0.00 | 0.00 |
| O | 0 | 2 | 0 | 0.05 | 0 | 0 |
| P | 0 | 30 | 0 | 0.89 | 0 | 0 |
| Q | 0 | 1 | 0 | 0.03 | 0 | 0 |

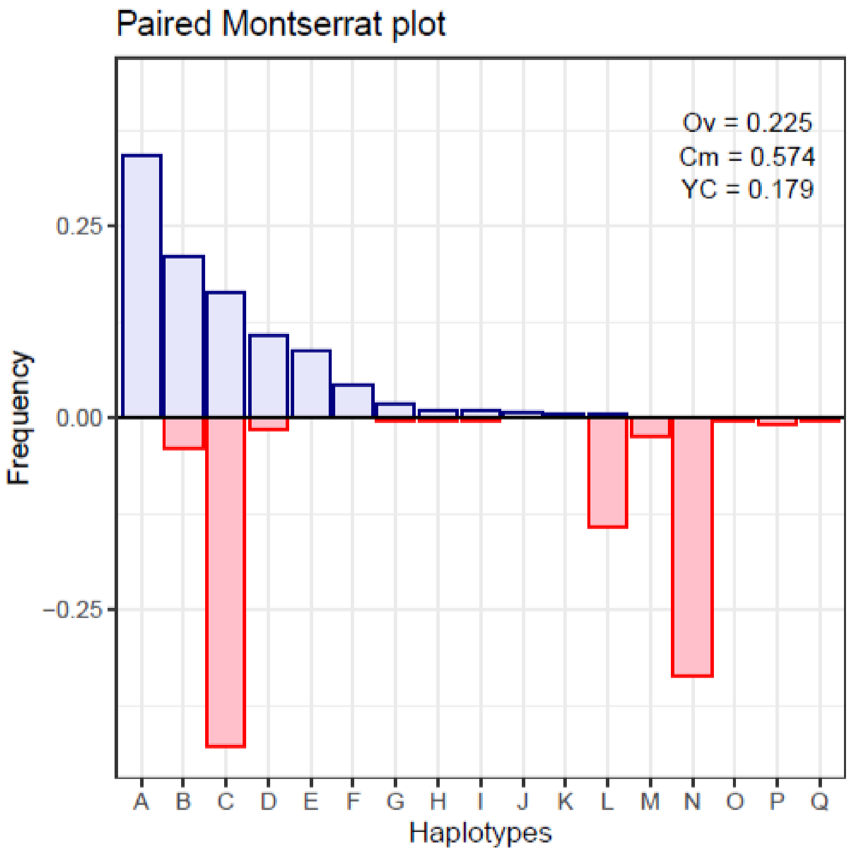


Figure S10: Simulated pair of index 3053.

| Hpl | nA | nB | pA | pB | Commons | Overlap |
|---|---|---|---|---|---|---|
| A | 1167 | 1 | 35.02 | 0.04 | 17.53 | 0.04 |
| B | 926 | 0 | 27.79 | 0.00 | 0.00 | 0.00 |
| C | 573 | 338 | 17.20 | 10.13 | 13.67 | 10.13 |
| D | 528 | 0 | 15.85 | 0.00 | 0.00 | 0.00 |
| E | 88 | 0 | 2.63 | 0.00 | 0.00 | 0.00 |
| F | 29 | 243 | 0.87 | 7.30 | 4.09 | 0.87 |
| G | 9 | 185 | 0.28 | 5.54 | 2.91 | 0.28 |
| H | 6 | 0 | 0.17 | 0.00 | 0.00 | 0.00 |
| I | 2 | 1239 | 0.07 | 37.16 | 18.62 | 0.07 |
| J | 2 | 414 | 0.07 | 12.41 | 6.24 | 0.07 |
| K | 1 | 0 | 0.03 | 0.00 | 0.00 | 0.00 |
| L | 1 | 4 | 0.03 | 0.12 | 0.08 | 0.03 |
| M | 0 | 111 | 0.00 | 3.34 | 0.00 | 0.00 |
| N | 0 | 9 | 0.00 | 0.28 | 0.00 | 0.00 |
| O | 0 | 637 | 0 | 19.1 | 0 | 0 |
| P | 0 | 141 | 0 | 4.24 | 0 | 0 |
| Q | 0 | 11 | 0 | 0.34 | 0 | 0 |

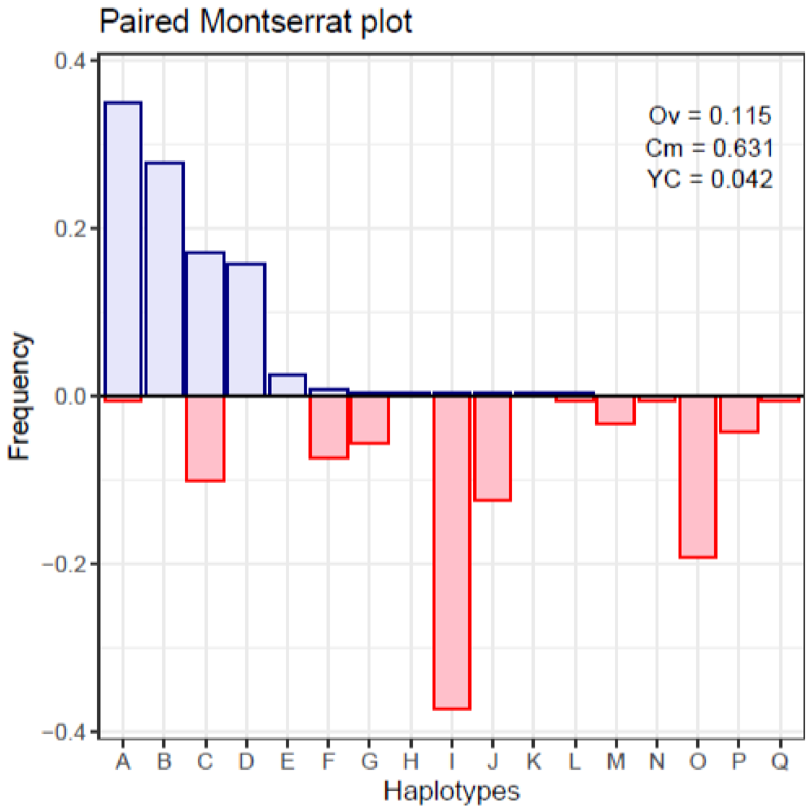


Figure S11: Simulated pair of index 5955.

| Hpl | nA | nB | pA | pB | Commons | Overlap |
|---|---|---|---|---|---|---|
| A | 2376 | 3108 | 47.53 | 62.16 | 54.85 | 47.53 |
| B | 1568 | 0 | 31.37 | 0.00 | 0.00 | 0.00 |
| C | 478 | 2 | 9.56 | 0.04 | 4.80 | 0.04 |
| D | 250 | 354 | 5.01 | 7.07 | 6.04 | 5.01 |
| E | 208 | 1184 | 4.16 | 23.69 | 13.93 | 4.16 |
| F | 43 | 0 | 0.86 | 0.00 | 0.00 | 0.00 |
| G | 34 | 0 | 0.68 | 0.00 | 0.00 | 0.00 |
| H | 26 | 0 | 0.51 | 0.00 | 0.00 | 0.00 |
| I | 8 | 5 | 0.15 | 0.10 | 0.13 | 0.10 |
| J | 4 | 6 | 0.07 | 0.12 | 0.10 | 0.07 |
| K | 4 | 0 | 0.07 | 0.00 | 0.00 | 0.00 |
| L | 1 | 0 | 0.03 | 0.00 | 0.00 | 0.00 |
| M | 0 | 2 | 0.00 | 0.04 | 0.00 | 0.00 |
| N | 0 | 28 | 0.00 | 0.55 | 0.00 | 0.00 |
| O | 0 | 216 | 0.00 | 4.33 | 0.00 | 0.00 |
| P | 0 | 1 | 0.00 | 0.02 | 0.00 | 0.00 |
| Q | 0 | 68 | 0.00 | 1.36 | 0.00 | 0.00 |
| R | 0 | 26 | 0.00 | 0.53 | 0.00 | 0.00 |

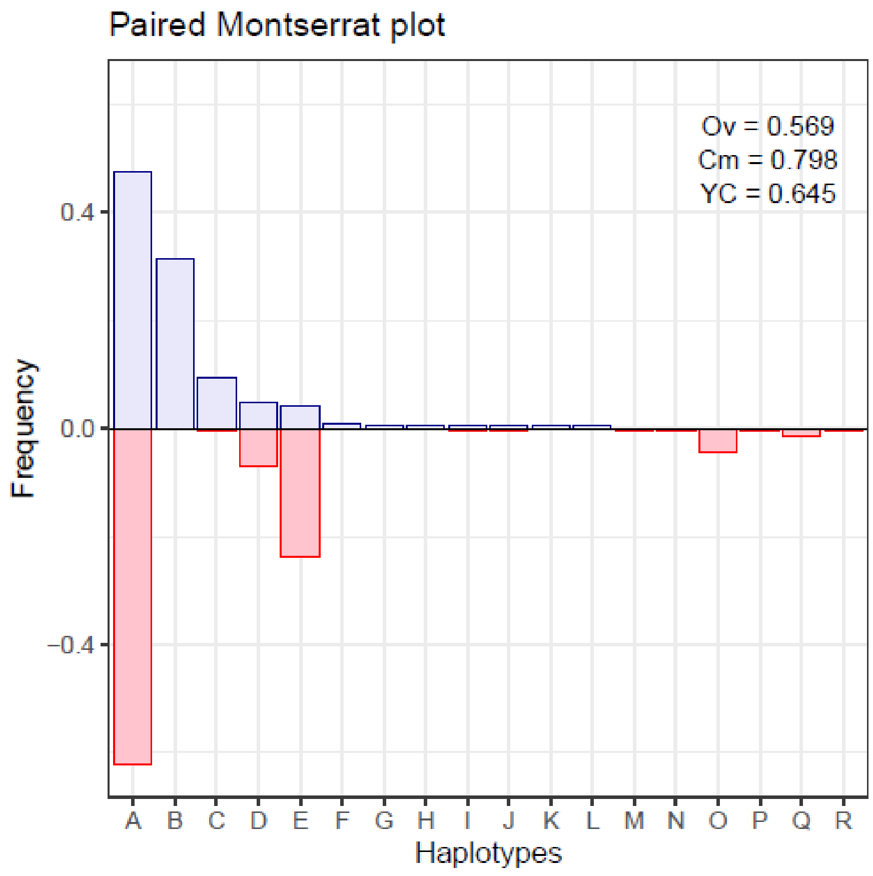


Figure S12: Simulated pair of index 1159.

| Hpl | nA | nB | pA | pB | Commons | Overlap |
|---|---|---|---|---|---|---|
| A | 5732 | 4936 | 57.32 | 49.36 | 53.34 | 49.36 |
| B | 1559 | 3222 | 15.59 | 32.22 | 23.91 | 15.59 |
| C | 1198 | 0 | 11.98 | 0.00 | 0.00 | 0.00 |
| D | 487 | 0 | 4.87 | 0.00 | 0.00 | 0.00 |
| E | 416 | 4 | 4.16 | 0.04 | 2.10 | 0.04 |
| F | 320 | 0 | 3.20 | 0.00 | 0.00 | 0.00 |
| G | 204 | 0 | 2.04 | 0.00 | 0.00 | 0.00 |
| H | 66 | 0 | 0.66 | 0.00 | 0.00 | 0.00 |
| I | 7 | 0 | 0.07 | 0.00 | 0.00 | 0.00 |
| J | 7 | 1 | 0.07 | 0.01 | 0.04 | 0.01 |
| K | 1 | 9 | 0.01 | 0.09 | 0.05 | 0.01 |
| L | 1 | 2 | 0.01 | 0.02 | 0.02 | 0.01 |
| M | 0 | 21 | 0.00 | 0.21 | 0.00 | 0.00 |
| N | 0 | 2 | 0.00 | 0.02 | 0.00 | 0.00 |
| O | 0 | 134 | 0.00 | 1.34 | 0.00 | 0.00 |
| P | 0 | 10 | 0.00 | 0.10 | 0.00 | 0.00 |
| Q | 0 | 1628 | 0.00 | 16.28 | 0.00 | 0.00 |
| R | 0 | 30 | 0.00 | 0.30 | 0.00 | 0.00 |

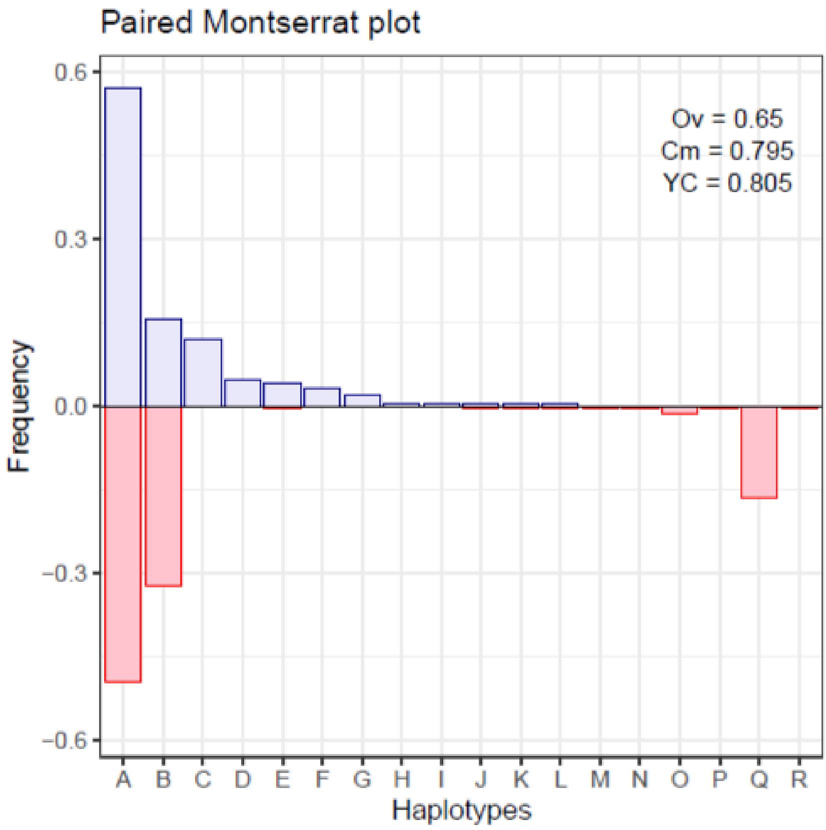


Figure S13: Simulated pair of index 2528.

| Hpl | nA | nB | pA | pB | Commons | Overlap |
|---|---|---|---|---|---|---|
| A | 3290 | 2354 | 65.79 | 47.07 | 56.43 | 47.07 |
| B | 662 | 0 | 13.25 | 0.00 | 0.00 | 0.00 |
| C | 558 | 1 | 11.17 | 0.03 | 5.60 | 0.03 |
| D | 283 | 20 | 5.66 | 0.41 | 3.04 | 0.41 |
| E | 72 | 0 | 1.43 | 0.00 | 0.00 | 0.00 |
| F | 64 | 17 | 1.28 | 0.34 | 0.81 | 0.34 |
| G | 40 | 1 | 0.80 | 0.03 | 0.42 | 0.03 |
| H | 12 | 0 | 0.24 | 0.00 | 0.00 | 0.00 |
| I | 10 | 114 | 0.20 | 2.29 | 1.25 | 0.20 |
| J | 6 | 59 | 0.13 | 1.18 | 0.66 | 0.13 |
| K | 1 | 0 | 0.03 | 0.00 | 0.00 | 0.00 |
| L | 1 | 488 | 0.02 | 9.75 | 4.89 | 0.02 |
| M | 0 | 734 | 0.00 | 14.69 | 0.00 | 0.00 |
| N | 0 | 33 | 0.00 | 0.66 | 0.00 | 0.00 |
| O | 0 | 3 | 0.00 | 0.06 | 0.00 | 0.00 |
| P | 0 | 1174 | 0.00 | 23.49 | 0.00 | 0.00 |

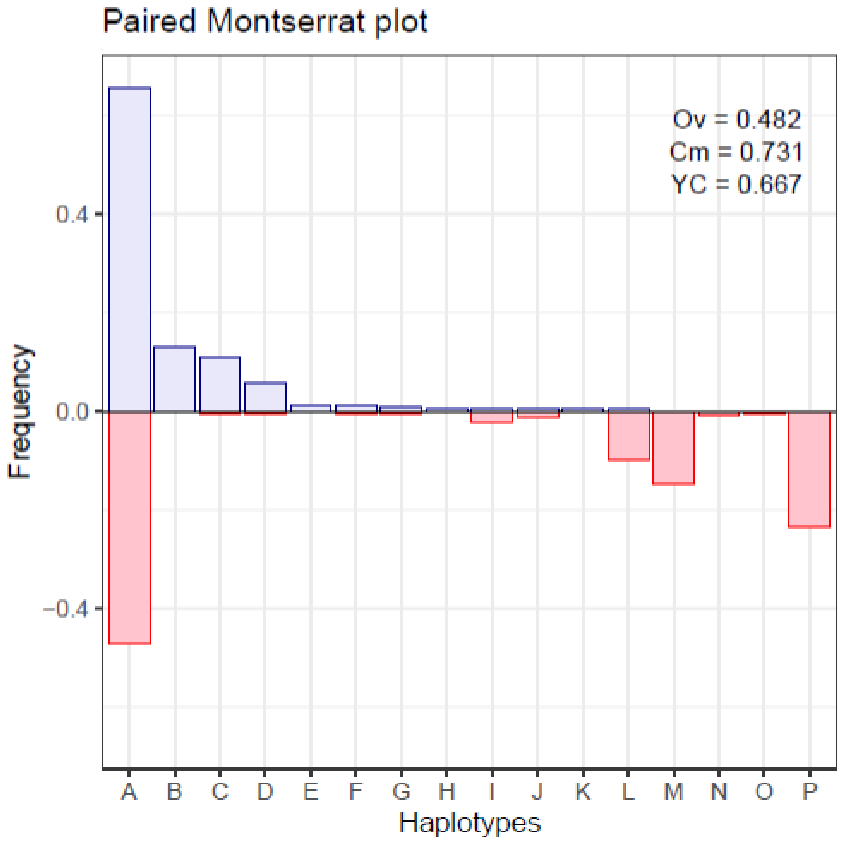


Figure S14: Simulated pair of index 345.

## A.4 Simulated evolution

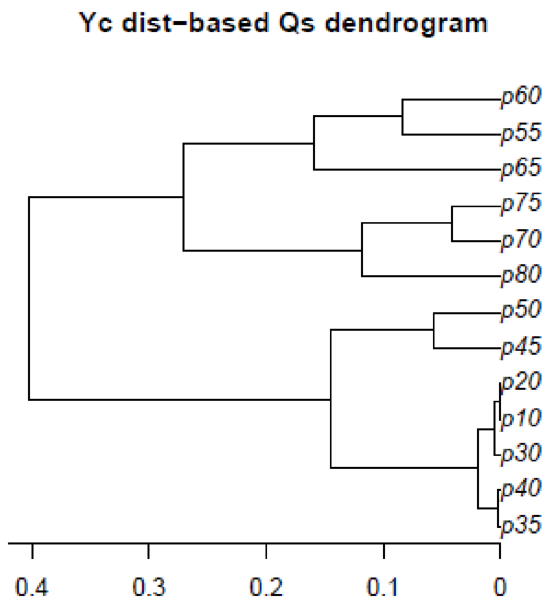


Figure S15: Quasispecies dendrogram based on Yue-Clayton distribution distances.

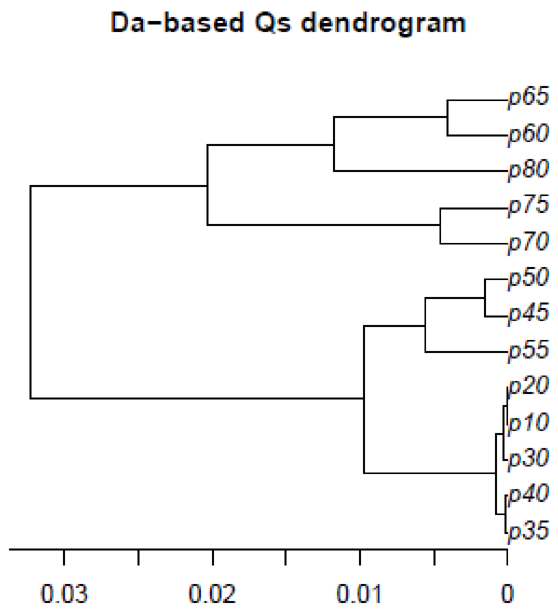


Figure S16: Quasispecies dendrogram based on $D_A$ genetic distances.

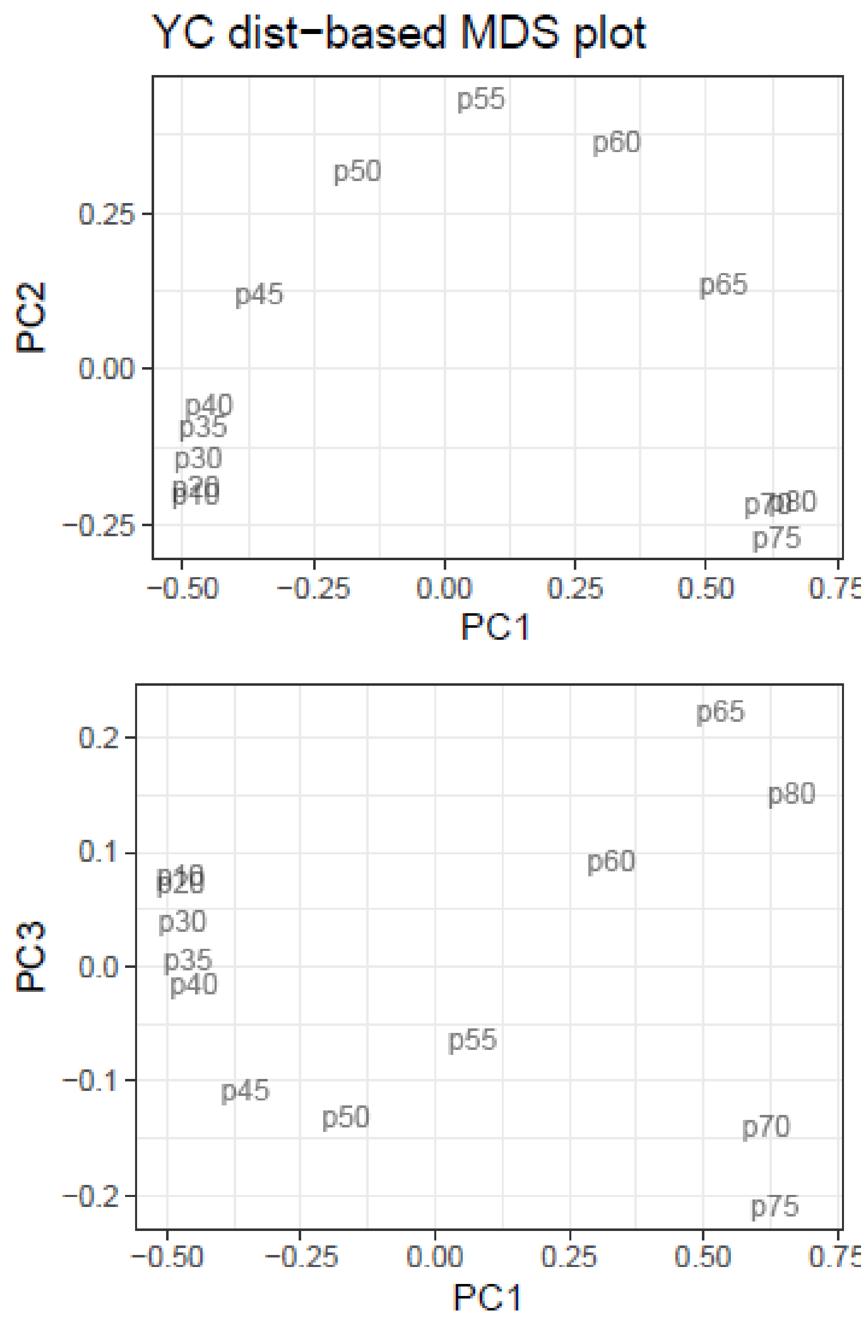


Figure S17: MDS plot based on Yue-Clayton distribution distances.

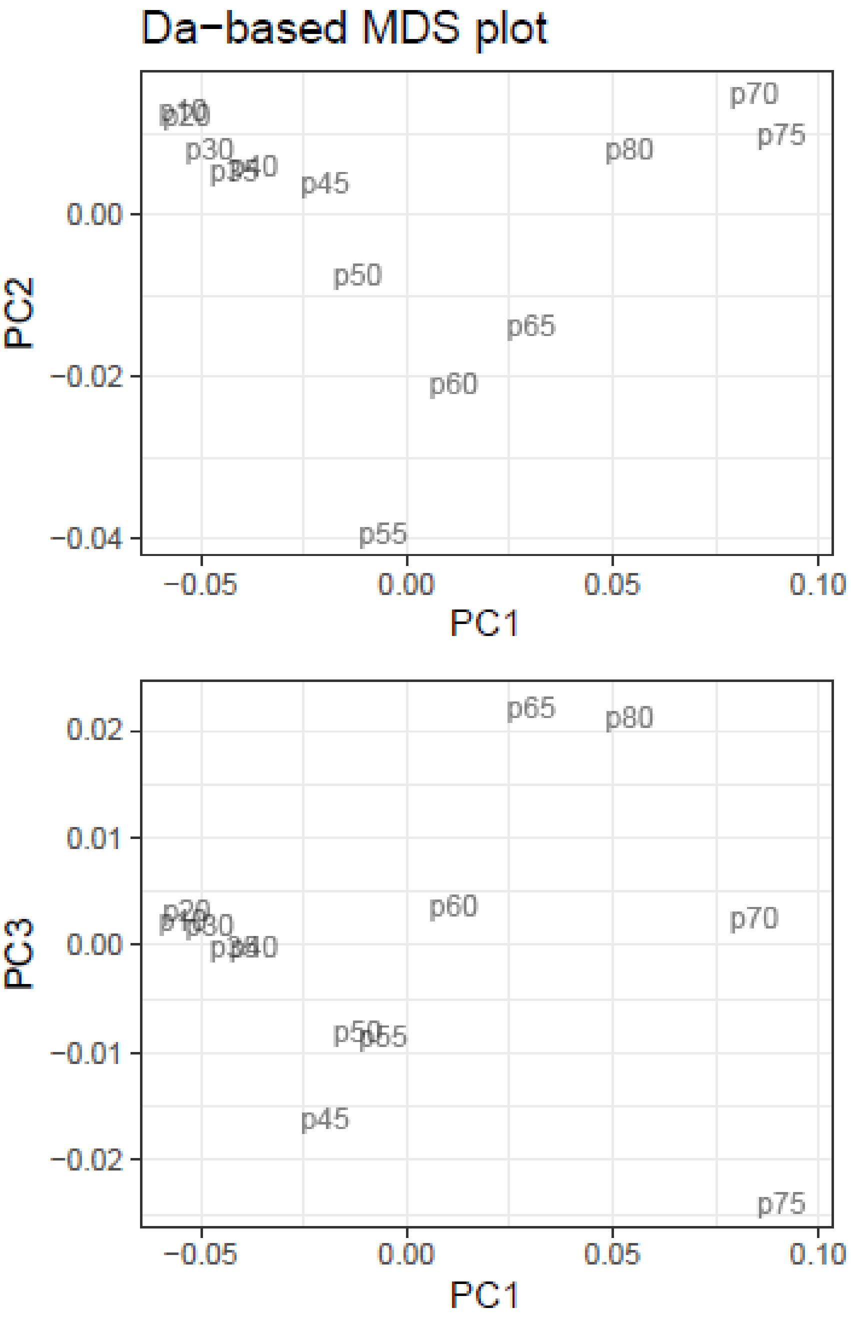


Figure S18: MDS plot based on $D_A$ genetic distances.

## B R code used in the simulation of pairs of quasispecies

```
library(tidyverse)

###  Yue & Clayton population overlap index
YueClayton_Overlap <- function(A,B)
{ pA <- A/sum(A)
  a <- sum(pA^2)
  pB <- B/sum(B)
  b <- sum(pB^2)
  d <- sum(pA*pB)
  return( d/(a+b-d) )
}

###  Commons
CommonReads <- function(A,B)
{ cm <- A>0 & B>0
  return( sum(A[cm]+B[cm])/sum(A+B) )
}

###  Raw overlap
MinOverlap <- function(A,B)
{ pA <- A/sum(A)
  pB <- B/sum(B)
  return( sum(pmin(pA,pB)) )
}

###  Generate a quasispecies distribution
GenQs <- function(n,m,p)
{
  ###  Sample from geometric distribution with p as parameter
  tb <- table(rgeom(20000,p))
  ###  Take a random sample of n frequencies
  idx <- sample(length(tb),n,replace=FALSE)
  ###  Distribution of m common haplotypes
  qsA <- rep(0.,m)
  ###  Sample which n haplotypes from m common to populate
  jdx <- sample(m,n,replace=FALSE)
  ###  Quasispecies frequency distribution
  qsA[jdx] <- round(tb[idx]/sum(tb[idx]),4)
  return(qsA)
}

###  Generate two random quasispecies and compute similarities
GenAndCompare <- function(n=12,m=20,p=0.2)
{ qsA <- GenQs(n,m,p)
```

```
  qsB <- GenQs(n,m,p)
  return(c(MinOverlap(qsA,qsB),
           CommonReads(qsA,qsB),
           YueClayton_Overlap(qsA,qsB),
           qsA,qsB))
}

###  Plot paired quasispecies
PlotQsPair <- function(qsA,qsB,is)
{ o <- order(qsA,decreasing=TRUE)
  ftbl <- data.frame(pA=qsA[o],pB=qsB[o])
  fl <- ftbl[,1]>0 | ftbl[,2]>0
  ftbl <- ftbl[fl,]
  ftbl[ftbl<0.005 & ftbl>0] <- 0.005
  ftbl$Hpl <- LETTERS[1:nrow(ftbl)]

  xlb <- nrow(ftbl)*0.9
  ymx <- max(c(ftbl[,1],ftbl[,2]))
  ylb <- ymx
  dflb <- data.frame(x=c(xlb,xlb,xlb),y=c(ylb*0.9,ylb*0.8,ylb*0.7),
                     lbl=paste(c('Ov =','Cm =','YC ='), round(is,3)))
  P <- ftbl %>%
    ggplot() +
      geom_col(aes(x=Hpl,y=pA),fill='lavender',col='navy') +
      geom_col(aes(x=Hpl,y=-pB),fill='pink',col='red') +
      geom_hline(yintercept=0) +
      ylim(-ymx,ymx) +
      labs(x='Haplotypes',y='Frequency',
           title='Paired Montserrat plot') +
      # geom_text(aes(x=x,y=y,label=lb),data=gmsg) +
      geom_text(aes(x=x,y=y,label=lbl),data=dflb) +
      theme_bw(base_size=12)
  print(P)
}

###  Plot k-th row of simulated data
PlotSimDt <- function(sdt,k)
{
  qsA <- sdt[k,(1:20)+3];
  qsB <- sdt[k,(1:20)+23];
  is <- sdt[k,1:3]
  PlotQsPair(qsA,qsB,is)
}

#-----------------------------------------------------------------------------#
```

```
set.seed(0925)
sdt <- t(replicate(10000,GenAndCompare()))
rownames(sdt) <- 1:nrow(sdt))
save(sdt,file='SimulatedPairs.RData')

#---------------------------------------------------------------------------#

sessionInfo()
R version 4.0.3 (2020-10-10)
Platform: x86_64-w64-mingw32/x64 (64-bit)
Running under: Windows 10 x64 (build 19043)

Matrix products: default

Random number generation:
 RNG:     Mersenne-Twister
 Normal:  Inversion
 Sample:  Rounding

locale:
[1] LC_COLLATE=Catalan_Spain.1252  LC_CTYPE=Catalan_Spain.1252
[3] LC_MONETARY=Catalan_Spain.1252 LC_NUMERIC=C
[5] LC_TIME=Catalan_Spain.1252

attached base packages:
[1] stats     graphics  grDevices utils     datasets  methods   base

other attached packages:
[1] forcats_0.5.1   stringr_1.4.0   dplyr_1.0.7     purrr_0.3.4
[5] readr_2.0.0     tidyr_1.1.3     tibble_3.1.3    ggplot2_3.3.5
[9] tidyverse_1.3.1

loaded via a namespace (and not attached):
 [1] Rcpp_1.0.7       cellranger_1.1.0 pillar_1.6.2     compiler_4.0.2
 [5] dbplyr_2.1.1     tools_4.0.2      digest_0.6.27    jsonlite_1.7.2
 [9] lubridate_1.7.10 lifecycle_1.0.0  gtable_0.3.0     pkgconfig_2.0.3
[13] rlang_0.4.11     reprex_2.0.1     cli_3.0.1        rstudioapi_0.13
[17] DBI_1.1.1        haven_2.4.3      xml2_1.3.2       withr_2.4.2
[21] httr_1.4.2       fs_1.5.0         generics_0.1.0   vctrs_0.3.8
[25] hms_1.1.0        grid_4.0.2       tidyselect_1.1.1 glue_1.4.2
[29] R6_2.5.0         fansi_0.5.0      readxl_1.3.1     farver_2.1.0
[33] tzdb_0.1.2       modelr_0.1.8     magrittr_2.0.1   backports_1.2.1
[37] scales_1.1.1     ellipsis_0.3.2   rvest_1.0.1      assertthat_0.2.1
[41] colorspace_2.0-2 labeling_0.4.2   utf8_1.2.2       stringi_1.7.3
[45] munsell_0.5.0    broom_0.7.9      crayon_1.4.1
```

```
#--------------------------------------------------------------------------#
```

## C R code to compute genetic distance

```
library(tidyverse)

###  Load simulated pairs and their similarities
load('SimulatedPairs.RData')
si <- sdt[,1:3]
colnames(si) <- c('Ov','Cm','YC')
m <- 20
n <- nrow(sdt)
Pa <- sdt[,(1:m)+3]
Pb <- sdt[,(1:m)+m+3]
colnames(Pa) <- colnames(Pb) <- LETTERS[1:m]

###  Matrix of distances between haplotypes
###  All haplotypes show a single substitution with respect to
###    the master of the first QS
D <- matrix(2,m,m)
D[1,] <- 1
D[,1] <- 1
diag(D) <- 0

###  Nucleotíde diversity and distance between populations
pop.dst <- function(k)
{ o <- order(Pa[k,],decreasing=TRUE)
  Dx <- t(Pa[k,o]) %*% D %*% Pa[k,o]
  Pc <- sort(Pb[k,],decreasing=TRUE)
  Dy <- t(Pc) %*% D %*% Pc
  Dxy <- t(Pa[k,o]) %*% D %*% Pb[k,o]
  Da <- Dxy - 0.5*(Dx+Dy)
  c(Dx,Dy,Dxy,Da)
}

pd <- t(sapply(1:n,pop.dst))
colnames(pd) <- c('Dx','Dy','Dxy','Da')

save(pd,file='GeneticDistance.RData')

#------------------------------------------------------------------------#
```

## D R code used in the treatment follow-up simulation

```
library(tidyverse)
library(ape)

set.seed(11112022)

nh <- 40
f0 <- c(0.999,rep(0.001/(nh-1),nh-1))

k_mstr_max <- 1.05
k_mstr_min <- 0.85
k_emer_max <- 1.25
k_emer_min <- 0.95
k_rare_max <- 2.5
k_rare_min <- 0.8
kf <- matrix(c(k_mstr_min,k_mstr_max,k_emer_min,k_emer_max,
               k_rare_min,k_rare_max),nrow=2)
rownames(kf) <- c('min','max')
colnames(kf) <- c('mstr','emer','rare')

ns <- 10

evol_step <- function(f,kf,nr)
{
  ### Evolve master
  f[1] <- f[1] * runif(1,kf[1,1],kf[2,1])
  ### Evolve emerging
  f[2] <- f[2] * runif(1,kf[1,2],kf[2,2])
  ### Evolve other haplotypes
  nc <- sample(2:nr,1)
  kdx <- sample(3:nh,nc,replace=FALSE)
  f[kdx] <- f[kdx] * runif(nc,kf[1,3],kf[2,3])

  ### Renormalize
  f <- f/sum(f)
  f
}

do_steps <- function(f,N,ct=kf,nr=ns)
{ for(i in 1:N)
    f <- evol_step(f,ct,nr)
  f
}

p10 <- do_steps(f0,10)
```

```
p20 <- do_steps(p10,10)
p30 <- do_steps(p20,10)
p35 <- do_steps(p30,5)
p40 <- do_steps(p35,5)
p45 <- do_steps(p40,5)
p50 <- do_steps(p45,5)
p55 <- do_steps(p50,5)
p60 <- do_steps(p55,5)
p65 <- do_steps(p60,5)
p70 <- do_steps(p65,5)
p75 <- do_steps(p70,5)
p80 <- do_steps(p75,5)

#----------------------------------------------------------------------#

###  Qs Hpl distributions plot

qs_set <- rbind(p10,p20,p30,p35,p40,p45,p50,p55,p60,p65,p70,p75,p80)
rownames(qs_set) <- c('p10','p20','p30','p35','p40','p45','p50','p55',
                      'p60','p65','p70','p75','p80')
colnames(qs_set) <- paste('h',sprintf('%02d',1:40),sep='')

P <- qs_set %>% data.frame() %>%
  rownames_to_column(var="Qs.ID") %>%
  pivot_longer(-Qs.ID,names_to="Hpl.ID",values_to="Freq") %>%
  # mutate(Pos=ifelse(Freq>0,'·','')) %>%
  ggplot() +
    geom_tile(aes(x=Hpl.ID,y=Qs.ID,fill=Freq)) +
    scale_fill_distiller(type = "seq",palette = "Greys",direction=1,
                         limits = c(0,1)) +
    # geom_text(aes(x=Hpl.ID,y=Qs.ID,label=Pos),size=6) +
    labs(title="Qs haplotype frequencies evolution",fill='') +
    theme_bw(base_size=14) +
    theme(axis.text.x = element_text(angle = 90, vjust = 0.5, hjust=1)) +
    theme(legend.position='bottom') +
    theme(legend.key.width = unit(1,"cm"))

pdf("QsHplDistributions.pdf",paper='a4',width=6.5,height=6)
print(P)
dev.off()

#----------------------------------------------------------------------#

YueClayton_Overlap <- function(A,B)
{ pA <- A/sum(A)
  a <- sum(pA^2)
```

```
  pB <- B/sum(B)
  b <- sum(pB^2)
  d <- sum(pA*pB)
  return( d/(a+b-d) )
}

Commons <- function(A,B)
{ cm <- A>0 & B>0
  return( sum(A[cm]+B[cm])/sum(A+B) )
}

MinOverlap <- function(A,B)
{ pA <- A/sum(A)
  pB <- B/sum(B)
  return( sum(pmin(pA,pB)) )
}

n <- nrow(qs_set)
Cm <- matrix(0,nrow=n,ncol=n)
rownames(Cm) <- colnames(Cm) <- rownames(qs_set)
Yc <- Ov <- Cm

for(i in 2:n)
{ for(j in 1:(i-1))
  {
    A <- qs_set[i,]
    B <- qs_set[j,]
    Yc[i,j] <- YueClayton_Overlap(A,B)
    Ov[i,j] <- MinOverlap(A,B)
    Cm[i,j] <- Commons(A,B)
  }
}
Yc <- Yc + t(Yc)
Ov <- Ov + t(Ov)
Cm <- Cm + t(Cm)

D.yc <- as.dist(1-Yc)
D.ov <- as.dist(1-Ov)
D.cm <- as.dist(1-Cm)

#----------------------------------------------------------------------#

###  Plot Qs dendrograms
pdf("QsDendrograms.pdf",paper='a4',width=5,height=5)

ddg <- hclust(D.yc,method='average')
```

```
plot(as.phylo(ddg))
title('Yc dist-based Qs dendrogram')
axisPhylo()

ddg <- hclust(D.ov,method='average')
plot(as.phylo(ddg))
title('Ov dist-based Qs dendrogram')
axisPhylo()

dev.off()

#------------------------------------------------------------------------#

###  Plot matrices of distances between Qs

ddg <- hclust(D.yc,method='average')
o <- ddg$order

P <- as.matrix(D.yc) %>% data.frame() %>%
  rownames_to_column(var="QsA") %>%
  pivot_longer(-QsA,names_to="QsB",values_to="Dist") %>%
  mutate(QsA=factor(QsA,levels=rownames(qs_set)[o]),
         QsB=factor(QsB,levels=rownames(qs_set)[o])) %>%
  ggplot() +
    geom_tile(aes(x=QsA,y=QsB,fill=Dist)) +
    scale_fill_gradient(trans='reverse',limits=c(1.,0.)) +
    labs(x='',y='',title='Yue-Clayton distances') +
    theme_bw(base_size=14) +
    theme(axis.text.x = element_text(angle = 90, vjust = 0.5, hjust=1)) +
    theme(legend.position='bottom') +
    theme(legend.key.width = unit(0.75,"cm"))

ddg <- hclust(D.ov,method='average')
o <- ddg$order

Q <- as.matrix(D.ov) %>% data.frame() %>%
  rownames_to_column(var="QsA") %>%
  pivot_longer(-QsA,names_to="QsB",values_to="Dist") %>%
  mutate(QsA=factor(QsA,levels=rownames(qs_set)[o]),
         QsB=factor(QsB,levels=rownames(qs_set)[o])) %>%
  ggplot() +
    geom_tile(aes(x=QsA,y=QsB,fill=Dist)) +
    scale_fill_gradient(trans='reverse',limits=c(1.,0.)) +
    labs(x='',y='',title='Distribution overlap distances') +
    theme_bw(base_size=14) +
    theme(axis.text.x = element_text(angle = 90, vjust = 0.5, hjust=1)) +
```

```
    theme(legend.position='bottom') +
    theme(legend.key.width = unit(0.75,"cm"))

ddg <- hclust(D.cm,method='average')
o <- ddg$order

pdf("QsHplDistributionDistances.pdf",paper='a4',width=5,height=6.5)
print(P)
print(Q)
dev.off()

#----------------------------------------------------------------------#

div_vals <- function(p)
{
  qff_top <- max(p)
  qff_mid <- sum(p[p>0.01])-max(p)
  qff_1   <- sum(p[p<=0.01])
  qff_0.1 <- sum(p[p<=0.001])

  return(c(qff_top,qff_mid,qff_1,qff_0.1))
}

qff <- t(apply(qs_set,1,div_vals))
colnames(qff) <- c('Master','Emerging','Pct1','Pct0.1')

P <- qff %>% data.frame() %>%
  rownames_to_column(var='Qs.ID') %>%
  pivot_longer(-Qs.ID,names_to='Fraction',values_to='Freq') %>%
  mutate(Fraction=factor(Fraction,
                    levels=rev(c('Pct0.1','Pct1','Emerging','Master')))) %>%
  ggplot() +
    geom_col(aes(x=Qs.ID,y=Freq,fill=Fraction),col='navy') +
    labs(x='',y='Fraction',title='QFF') +
    theme_bw(base_size=14) +
    theme(legend.position='bottom')

pdf("QFF_barplot.pdf",paper='a4',width=5,height=5)
print(P)
dev.off()

#----------------------------------------------------------------------#

###  Ov dist-based Multidimensional Scaling

svd <- cmdscale(D.ov,k=3,eig=TRUE,add=TRUE)
```

```
svd.xyz <- svd$points %>% data.frame() %>%
  rownames_to_column(var='ID')

P <- svd.xyz %>%
  ggplot() +
    geom_text(aes(x=X1,y=X2,label=ID),alpha=0.5) +
    xlim(range(svd.xyz$X1)*1.05) +
    labs(x='PC1',y='PC2',title='Ov dist-based MDS plot') +
    theme_bw(base_size=14)

Q <- svd.xyz %>%
  ggplot() +
    geom_text(aes(x=X1,y=X3,label=ID),alpha=0.5) +
    xlim(range(svd.xyz$X1)*1.05) +
    labs(x='PC1',y='PC3') +
    theme_bw(base_size=14)

R <- svd.xyz %>%
  ggplot() +
    geom_text(aes(x=X2,y=X3,label=ID),alpha=0.5) +
    xlim(range(svd.xyz$X2)*1.05) +
    labs(x='PC2',y='PC3') +
    theme_bw(base_size=14)

library(gridExtra)
pdf('OvDist.MDS.Plot.pdf',paper='a4',width=4.5,height=10.5)
print(grid.arrange(P,Q,R,ncol=1))
dev.off()

#------------------------------------------------------------------------#

###  YC dist-based Multidimensional Scaling

svd <- cmdscale(D.yc,k=3,eig=TRUE,add=TRUE)

svd.xyz <- svd$points %>% data.frame() %>%
  rownames_to_column(var='ID')

P <- svd.xyz %>%
  ggplot() +
    geom_text(aes(x=X1,y=X2,label=ID),alpha=0.5) +
    xlim(range(svd.xyz$X1)*1.05) +
    labs(x='PC1',y='PC2',title='YC dist-based MDS plot') +
    theme_bw(base_size=14)
```

```
Q <- svd.xyz %>%
  ggplot() +
    geom_text(aes(x=X1,y=X3,label=ID),alpha=0.5) +
    xlim(range(svd.xyz$X1)*1.05) +
    labs(x='PC1',y='PC3') +
    theme_bw(base_size=14)

R <- svd.xyz %>%
  ggplot() +
    geom_text(aes(x=X2,y=X3,label=ID),alpha=0.5) +
    xlim(range(svd.xyz$X2)*1.05) +
    labs(x='PC2',y='PC3') +
    theme_bw(base_size=14)

library(gridExtra)
pdf('YcDist.MDS.Plot.pdf',paper='a4',width=4.5,height=10.5)
print(grid.arrange(P,Q,R,ncol=1))
dev.off()

#------------------------------------------------------------------------#

###  Matrix of pair-wise haplotype nucleotide distances
D <- matrix(2,nrow=nh,ncol=nh)
D[1,] <- 1
D[,1] <- 1
diag(D) <- 0

###  Diversitat nucleotídica i distància entre poblacions

Dx <- numeric(nrow(qs_set))
names(Dx) <- rownames(qs_set)
Dxy <- Da <- matrix(0,nrow=nrow(qs_set),ncol=nrow(qs_set))
rownames(Dxy) <- colnames(Dxy) <- rownames(qs_set)
rownames(Da) <- colnames(Da) <- rownames(qs_set)

for(i in 1:nrow(qs_set))
  Dx[i] <- t(qs_set[i,]) %*% D %*% qs_set[i,]

for(i in 2:nrow(qs_set))
{ for(j in 1:(i-1))
  { Dxy[i,j] <- t(qs_set[i,]) %*% D %*% qs_set[j,]
    Da[i,j] <- Dxy[i,j] - 0.5*(Dx[i]+Dx[j])
  }
}
Dxy <- Dxy + t(Dxy)
Da <- Da + t(Da)
```

```
###  Plot Da-based Qs dendrogram
pdf("DaBased_QsDendrogram.pdf",paper='a4',width=5,height=5)

ddg <- hclust(as.dist(Da),method='average')
plot(as.phylo(ddg))
title('Da-based Qs dendrogram')
axisPhylo()

dev.off()

###  Da-based Multidimensional Scaling

svd <- cmdscale(Da,k=3,eig=TRUE,add=TRUE)

svd.xyz <- svd$points %>% data.frame() %>%
  rownames_to_column(var='ID')

P <- svd.xyz %>%
  ggplot() +
    geom_text(aes(x=X1,y=X2,label=ID),alpha=0.5) +
    xlim(range(svd.xyz$X1)*1.05) +
    labs(x='PC1',y='PC2',title='Da-based MDS plot') +
    theme_bw(base_size=14)

Q <- svd.xyz %>%
  ggplot() +
    geom_text(aes(x=X1,y=X3,label=ID),alpha=0.5) +
    xlim(range(svd.xyz$X1)*1.05) +
    labs(x='PC1',y='PC3') +
    theme_bw(base_size=14)

R <- svd.xyz %>%
  ggplot() +
    geom_text(aes(x=X2,y=X3,label=ID),alpha=0.5) +
    xlim(range(svd.xyz$X2)*1.05) +
    labs(x='PC2',y='PC3') +
    theme_bw(base_size=14)

library(gridExtra)
pdf('Da.MDS.Plot.pdf',paper='a4',width=4.5,height=10.5)
print(grid.arrange(P,Q,R,ncol=1))
dev.off()

#------------------------------------------------------------------------#
```
